# Natural and Synthetic Pyrethrins Act as Feeding Deterrents against the Black Blowfly, *Phormia regina* (Meigen)

**DOI:** 10.3390/insects13080678

**Published:** 2022-07-27

**Authors:** Takeshi Kojima, Seiji Yamato, Shinichi Kawamura

**Affiliations:** 1 Health & Crop Sciences Research Laboratory, Sumitomo Chemical Co., Ltd., 2-1 Takatsukasa 4-Chome, Takarazuka, Hyogo 665-8555, Japan; yamatos2@sc.sumitomo-chem.co.jp (S.Y.); kawamuras4@sc.sumitomo-chem.co.jp (S.K.); 2 Department of Biology, Graduate School of Science, Kobe University, 1-1 Rokkodai-cho, Nada-ku, Kobe 657-8501, Japan

**Keywords:** pyrethrins, pyrethrum, feeding deterrent, antifeedant, insecticide, *Phormia regina*

## Abstract

**Simple Summary:**

Pyrethrum is a botanical insecticide derived from pyrethrum flowers. Feeding deterrence caused by pyrethrum has been noted for several insect species. However, it is unclear whether the deterrent property results from a single component or from a combination of the six insecticidal active ingredients, known as pyrethrins. Here, we determined the feeding deterrence of natural pyrethrins and their two main components (pyrethrins I and II) on the blowfly, *Phormia regina*, in a dual-choice feeding assay. We found that natural pyrethrins and synthetic pyrethrins I/II in sucrose solution induced feeding deterrence at a concentration 16 times lower than the lowest concentration at which insecticidal action occurs. These results demonstrate that pyrethrins act as a feeding deterrent at sub-lethal concentrations. At the deterring concentration, feeding bouts were interrupted by intensive grooming of the proboscis, suggesting that pyrethrins acted instantly on the oral gustatory system of flies. The potent feeding deterrence of pyrethrins may provide effective protection for pyrethrum plants by rapidly deterring insects from feeding before insecticidal activities occur.

**Abstract:**

Pyrethrum is a botanical insecticide derived from pyrethrum flowers. Feeding deterrence caused by pyrethrum has been reported in several sucking insects; however, there is no account of the cause of deterrence—whether from a single component or the combination of six active ingredients, called pyrethrins. We determined the feeding deterrence of natural pyrethrins, their two main components (pyrethrins I and II), and pyrethroid insecticides on the blowfly, *Phormia regina*. In a dual-choice feeding assay that minimized tarsal contact with food sources but allowed feeding through proboscises, natural pyrethrins, synthetic pyrethrins I/II, and allethrin were observed to induce deterrence at a concentration 16 times lower than the lowest concentration at which the knockdown rate increased. Feeding bouts were interrupted by intensive grooming of the proboscis at the deterring concentration, but no such grooming was observed to occur while feeding on the unpalatable tastants—NaCl, quinine, and tartaric acid. The underlying mode of action for the feeding deterrence of pyrethrins at sub-lethal concentrations probably occurs on the fly oral gustatory system, while differing from that of unpalatable tastants. The potent feeding deterrence of pyrethrins may provide effective protection for pyrethrum plants by rapidly deterring insects from feeding, before insecticidal activities occur.

## 1. Introduction

**Figure 1:**
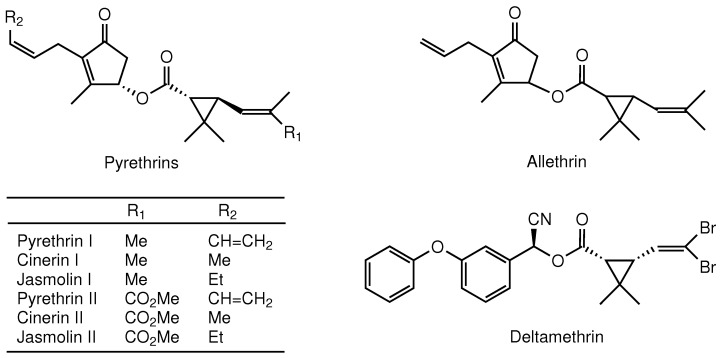
Chemical structures of the neurotoxic insecticides tested in dual-choice feeding assays.

Pyrethrum is a fast-acting, powerful insecticide derived from the flowers of pyrethrum daisy, *Tanacetum cinerariifolium* Sch. Bip. Owing to its broad-spectrum efficacy and low environmental and mammalian toxicity, pyrethrum has been widely used in agricultural, veterinary, and indoor pest-control products [1,2]. The active ingredients of pyrethrum, the six closely related insecticidal esters known as pyrethrins (Figure 1), accumulate in the aboveground parts of *T. cinerariifolium* plants [1,3]. Pyrethrins and pyrethroids, the synthetic derivatives of pyrethrins, exert their insecticidal effect by modulating the activities of voltage-gated sodium channels in the insect’s nervous system [4,5,6,7]. Lately, the use of synthetic pesticides has come under increasingly stringent regulations worldwide, and organic and natural pesticides have garnered attention as alternatives [8,9]. Therefore, it is important to deepen our understanding of the biological activities of pyrethrins for the efficient and sustainable use of pyrethrum.

Genomic and transcriptomic studies have identified the key enzymes involved in pyrethrin biosynthesis and have elucidated a considerable part of their biosynthetic pathways [10]. Pyrethrin biosynthetic genes are primarily expressed in the ovary during the pre-flowering stage and pyrethrin accumulation is induced by mechanical wounding and volatile organic compounds in the vegetative tissues. These regulatory mechanisms suggest pivotal roles of pyrethrins as endogenous defense molecules against insect herbivores. It has been found that the western flower thrips, *Frankliniella occidentalis,* that infest pyrethrum flowers avoid feeding on pyrethrin-treated leaves at those concentrations of pyrethrins that were close to the actual pyrethrin content in pyrethrum leaves [11]. This finding supports the belief that pyrethrins accumulated in plant tissues protect the pyrethrum plant by deterring insects from feeding on the plant. This deterrence is caused by the oral ingestion of pyrethrins prior to the expression of insecticidal actions. Similar feeding avoidance behaviors have been reported in whiteflies and aphids [12]. However, in the leaf-disk assays performed in these studies, the legs of the sucking insects were continuously in direct contact with the pyrethrin-coated leaf surfaces. This could elicit a repellent action, referred to as contact irritation, based on tarsal contact with pyrethrins [13,14,15], before any effect via ingestion occurs. In addition, these studies determined the feeding deterrence of pyrethrum oil obtained from an extract of dried and ground pyrethrum flower heads. The crude extracts of pyrethrum flowers contain a variety of terpenes at a concentration of approximately 10% [1,3]. Since phytoterpenes generally have the potential for repellent action in the vapor phase [16], constituents other than pyrethrins might have caused the decrease in feeding activity. Several pyrethroid insecticides have been shown to induce feeding deterrence in the larvae of lepidopteran [17,18,19,20] and coleopteran species [21,22,23], neither of which have tarsal chemoreceptors, indicating that the antifeedant activity is induced by the oral intake of the synthetic insecticides. However, there is no direct evidence indicating whether the feeding deterrence in insects is caused by any one or a combination of the six pyrethrin components of pyrethrum.

*Drosophila melanogaster* and blowflies are excellent model organisms for studying the neurophysiological basis of feeding behavior and taste perception [24,25,26,27]. Several established feeding assays, including conventional dual/multiple-choice feeding [28,29], the proboscis extension response [30,31,32], and capillary feeding assays [33,34,35], enable us to quantify ingestion by flies and to assess attraction or aversion to a soluble chemical mixed in a simple artificial diet. Owing to these assay systems, feeding deterrence has been demonstrated for various natural and synthetic compounds in flies, some of which can be used as a positive control for salty, bitter, and sour tastants [24,27,28,36,37]. Here, we examined the feeding deterrence of natural pyrethrins and synthetic pyrethrins I and II against *Phormia regina* (Meigen), a common blowfly species. To this end, dual-choice feeding assays were performed to minimize fly tarsal contact with the food source while allowing them to feed via their proboscises. The antifeedant activities of natural and synthetic pyrethrins were compared with those of two pyrethroid insecticides—allethrin and deltamethrin—and also with those of salty, bitter, and sour compounds exhibiting deterrence against flies.

## 2. Materials and Methods

### 2.1. Fly Handling and Rearing

Blowflies (*P. regina*), originally obtained from the laboratory of Prof. M. Ozaki at Kobe University, were reared in our laboratory at 21 ± 1 °C and 70% RH under 16-h light/8-h dark cycles. Larvae were fed on pig liver. Newly emerged adults were reared in cages measuring 21 × 21 × 28 cm^3^ (height × width × depth) and were fed 0.1 M sucrose solution. We used 4–10 day-old unsexed adult flies in all experiments.

### 2.2. Chemicals

We tested a pyrethrum extract (CAS RN^®^: 8003-34-7, Fujifilm Wako Pure Chemical, Osaka, Japan) that included the six insecticidal pyrethrins and constituted 95.7% of the purified extract by weight; this could be treated as natural pyrethrins. Pyrethrins I and II, the major components of pyrethrins, were synthesized by the esterification of (S)-pyrethrolone with (+)-trans-chrysanthemic acid and (+)-trans-pyrethric acid, respectively [38]. Their purities (I: 99.4%; II: 96.9%) were determined using liquid chromatography. Butylated hydroxytoluene (BHT) was added to these compounds (1% *w*/*w*) to prevent oxidation. A Type I pyrethroid, allethrin (a mixture of cis and trans isomers, 95.9% purity; Sigma-Aldrich Chemical Corp., St. Louis, MO, USA) and a Type II pyrethroid, deltamethrin (99.5% purity; Kanto Chemical, Tokyo, Japan) were also tested. These natural and synthetic insecticides were all prepared as 100 mM stock solutions with dimethyl sulfoxide (DMSO; ≥99% purity; FUJIFILM Wako Pure Chemical, Osaka, Japan) and stored in a freezer at −20 °C until further use. NaCl (99.9% purity; Nacalai Tesque, Kyoto, Japan), quinine hydrochloride dihydrate (99.8% purity; FUJIFILM Wako Pure Chemical), and L-(+)-tartaric acid (99.9% purity; Sigma-Aldrich Chemical), which are salty, bitter, and sour tastants, respectively, were used as positive controls for taste deterrence. Sucrose (FUJIFILM Wako Pure Chemical) was used as feeding stimulant.

### 2.3. Evaluation of Feeding Deterrence

Feeding deterrence was evaluated using dual-choice feeding assays where flies were placed in a cage with a choice between one food source containing 100 mM sucrose (control solution) and another containing a test compound. For experiments with insecticidal compounds, the stock solutions in DMSO were diluted using a 100 mM sucrose. To avoid any unintended effect on feeding, DMSO was added in the same ratio to each control solution. The highest concentration of all insecticides was 1 mM and, accordingly, the test solution contained 1% DMSO. We prepared 16- or 4-fold serial dilutions of the test solutions to evaluate the function of dose. Water-soluble tastants, NaCl, quinine, and tartaric acid, were dissolved in a DMSO-free sucrose solution for comparison with the sucrose controls.

The 40–100 starved flies that had been provided water ad libitum for 20–24 h were released into a cage measuring 21 × 21 × 28 cm^3^ (height × width × depth). Two to six cages were placed in an incubator (LPH-241PFD-SP, Nippon Medical & Chemical Instruments Co. Ltd., Osaka, Japan) at 24 ± 1 °C and 70 ± 5% RH under light conditions. Two aluminum dishes, each with a diameter of 4.7 cm and a depth of 0.9 cm, weighing 0.46 ± 0.00 g (mean ± sd, 12 dishes), were filled with cotton pads (5 × 6 × 0.4 cm^3^, height × width × thickness; 0.52 ± 0.00 g, mean ± sd, 12 pads), which were then soaked with 10 mL of test or control solution. To avoid direct tarsal contact with the test and control solutions, the dishes were covered with circular polyester netting (mesh size: 1.5 mm). The two food dishes were placed 5 cm apart in the center of each cage after measuring the initial weights of the dishes, and the 5 h-long feeding experiments were initiated. The number of knocked-down flies, defined as flies lying on their side or back and unable to right themselves, was counted at the end of the experiments and the food dishes were then weighed. A total of 5 replicates were performed for each compound and each concentration.

### 2.4. Calculation of Food Intake

Food intake was measured as the difference in the weight of the dishes before and after testing. Weight loss was normalized for evaporation and was determined by weighing each of the paired dishes containing 100 mM sucrose solution under the same experimental conditions. The amount of water that evaporated from a control sucrose solution in the 5 h feeding time was approximately 0.69 g, regardless of the DMSO content (with 1% DMSO: 0.69 ± 0.05 g, mean ± sd, *n* = 8; without DMSO: 0.69 ± 0.08 g, *n* = 8). The intake amount for each food dish was obtained by subtracting the averaged natural evaporation (0.69 g) from the weight loss in the food dishes in each trial. The resultant intake values of the sucrose solution ranged from 1.06 g (*n* = 44 flies) to 3.39 g (*n* = 98 flies); this was roughly proportional to the number of flies released into the cage. An electronic balance (PM4000, METTLER TOLEDO, Columbus, OH, USA), with an accuracy within 10 mg, provided sufficient resolution to measure the potential decrease in food intake owing to the effects of the test compounds.

### 2.5. Analysis of Feeding Deterrence and Knockdown (KD) Activity

In many studies that evaluated attraction or aversion to a test compound (mixed in food) in a choice feeding assay, feeding deterrence of the test compound was demonstrated by the decrease in feeding preference for the test compound with respect to that for the control food [11,12,37,39]. We obtained a preference index (PI) that was calculated according to the equation (It − Ib)/(It + Ib), where It and Ib represent the intake of test and control solutions in each experiment, respectively. PI values of 1.0 and −1.0 indicated complete preference for and deterrence against the test chemical, respectively, whereas a PI of 0.0 indicated no bias between the two food choices. In the case of a negative value of food intake, often observed in complete deterrence, the PI values were calculated assuming that the food intake was 0, to ensure that the values lay within a normalized range. To evaluate the insecticidal effects of pyrethrins and pyrethroids, the KD rate was defined as the number of knocked-down flies divided by the number of flies released in the cage.

### 2.6. Baseline Validation of a Choice-Feeding Assay

DMSO, used to dissolve liposoluble pesticides in the sucrose solution, was maintained in a constant ratio to the molar concentration (percent volume) of the compounds. DMSO is toxic to many organisms, including insects, but the lethality depends not only on the concentration of DMSO in solutions but also on the species used for experiments and the test methods employed [40,41,42,43,44]. To evaluate the impact of DMSO on the feeding behavior of the blowflies, we performed a series of dual-choice feeding assays, each with a pair of identical sucrose and DMSO solutions, with varying DMSO concentrations from 1 M to 10^−5^ M through the series. We analyzed the values of individual food intake per hour, which were obtained by dividing the total volume of food intake by the number of flies released into the cage and by the test period and KD rate as a function of the concentration of DMSO. Additionally, the potential feeding deterrence of DMSO for the blowflies was investigated by a feeding test using a 1% DMSO sucrose solution and a DMSO-free sucrose solution. Similarly, the potential feeding deterrence of BHT was investigated through a feeding test using a 20 μM BHT sucrose solution and a BHT-free sucrose solution, both of which contained 1% DMSO. The BHT concentration, at 20 μM, was slightly higher than the actual content of 14.9 μM in the test solution with 1 mM synthetic pyrethrins.

### 2.7. Determination of Effective Concentrations and Estimation of Individual Intake

For concentration–response data of the test compounds, we determined the concentrations at which feeding deterrence/KD effect occurred. Feeding deterrence was established when the mean values of PIs were significantly less than 0; KD activity was established when the mean KD rates were significantly higher than a KD rate of 0%. We then specified the minimum effective concentration (MEC) for each compound; MEC was defined as the lowest concentration of a test compound at which an antifeedant or a KD effect occurred.

The strength of an insecticidal effect is often quantified by the dose of the compound that induces a certain insecticidal effect. Notably, the quantities of a compound that need to be ingested to induce feeding deterrence and KD effects are fundamentally different, even at the same MECs, since the intake of the sucrose solution decreases upon feeding deterrence. Thus, to compare the intake of a compound that induced feeding deterrence or a KD effect with other test compounds, we assumed that all the flies ingested the test sucrose solution equally. The individual intake of a compound was estimated by dividing the weight of the test compound, present at MEC in the volume of the sucrose solution ingested, by the number of flies released into the cage.

### 2.8. Characterization of Feeding Deterrence of Pyrethrins and Pyrethroids

In a test for natural pyrethrins, we observed that once the blowflies had fed on the treated sucrose solution, they stopped feeding and initiated intensive grooming of their proboscises with their forelegs (Video S1). To examine the correlation between feeding-induced proboscis grooming with feeding behavior and insecticidal effects, blowfly feeding behavior in a dual-feeding situation was recorded by a digital camera (COOLPIX A900, Nikon, Tokyo, Japan). To obtain the individual feeding history, 15 starved flies with distinguishable markings on their thorax were tested in each trial. These tests were repeated thrice for each compound. The initial feeding behavior was recorded in the first 30 min of the 5 h test. The number of knocked-down flies was counted at the end of the test.

Video recordings were analyzed using the Microsoft Photos application. Fly visits followed by feeding at an untreated or treated food dish were identified and the history of food searches was obtained for each fly. To examine the influence of the test compound ingested in the first feeding on subsequent food searches, we counted the number of visits to a food dish by individuals who first visited a treated food dish. For each compound, the rate at which grooming of the proboscis occurred, during visits to a treated food dish, was calculated.

### 2.9. Statistical Analysis

Statistical analyses were performed using R, version 4.1.2, and figures were designed with Igor Pro 6.3.7.2 (WaveMetrics, Lake Oswego, OR, USA). The averages of PIs and KD rates obtained for each compound were compared with *p* = 0 and a KD rate of 0%, respectively, by one-sample *t*-tests, with Bonferroni correction for multiple comparisons. Estimated individual intakes at MECs across compounds were compared with one-sample *t*-tests with Bonferroni correction. The effect of DMSO concentrations on the averages of individual food intake was compared with one-way ANOVA. The Tukey’s method was used for multiple comparisons of the average number of visits to a food dish within a group. Multiple comparisons of the frequencies of proboscis grooming and KD rates across compounds were made using the Fisher’s exact test with the *p*-values adjusted using Holm’s method.

## 3. Results

### 3.1. Baseline Validation of the Choice-Feeding Assay

**Figure 2:**
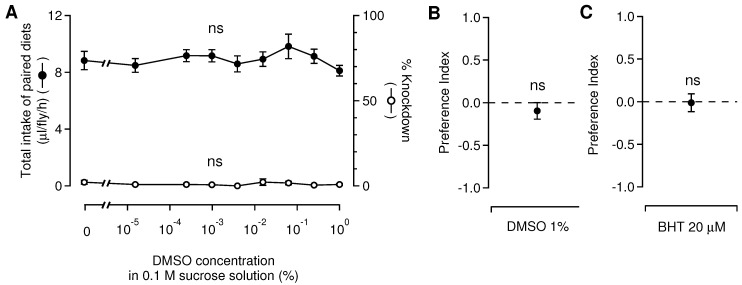
Baseline validation of a dual-choice feeding assay: impact of DMSO and BHT on the feeding preference of blowflies. (**A**) The mean values of individual food intake and knockdown (KD) rates are shown as a function of DMSO concentration. An increased concentration of DMSO did not change the average values of individual food intake (ANOVA, df = 8, F = 0.636, *p* = 0.742) and did not increase the KD rate (one sample *t*-test vs a KD rate of 0%, *p* > 0.563). (**B**) 1% DMSO in sucrose solution did not reduce the average of PIs from PI = 0 (one sample *t*-test, *p* = 0.424). (**C**) 20 µM of BHT, the amount contained in the initial concentration (1 mM) of synthetic pyrethrins, did not change the average of PIs (one sample *t*-test, *p* = 0.918). Error bars indicate standard error of the mean (*n* = 5 repetitions).

When the blowflies fed on sucrose solutions with varying concentrations of DMSO, the average values of individual food intake did not change with changes in DMSO concentration (Figure 2A). A higher DMSO concentration did not increase the KD rate (Figure 2A). A DMSO concentration of 1% in sucrose solution, corresponding to the maximum DMSO content in a test solution containing the highest concentration (1 mM) of insecticidal components, did not reduce the average value of PI (Figure 2B). At 20 µM of BHT, the amount present in the highest concentration (1 mM) of synthetic pyrethrins, no change was observed in the average PIs (Figure 2C). These results confirmed that DMSO added to sucrose solutions of pyrethrins and pyrethroids did not disrupt the baseline feeding preference in dual-choice feeding assays, and that BHT added to the synthetic pyrethrins at a constant ratio also did not cause feeding deterrence.

### 3.2. Evaluation of Feeding Deterrence

**Figure 3:**
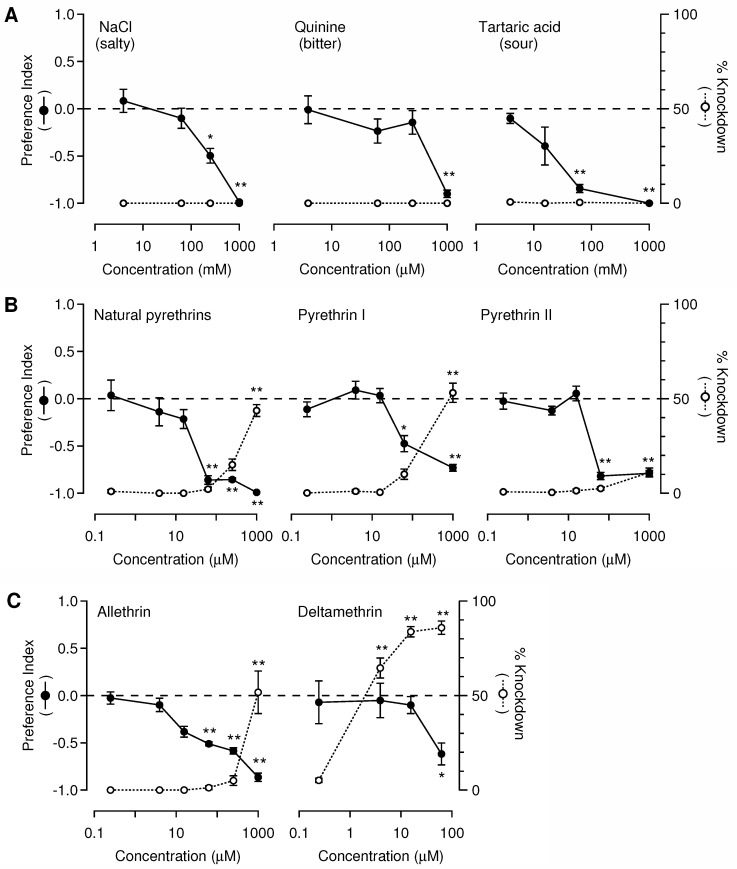
Feeding deterrence of neurotoxic insecticides and known anti-tastants in dual-choice feeding assays. Paired-choice experiments revealed a preference of blowflies for 0.1 mM sucrose solution over 0.1 mM sucrose solution mixed with various concentrations of test compounds. Preference indexes (PIs) and knockdown (KD) rates are indicated as a function of treatment concentrations for: (**A**) salty, bitter, and sour tastants; (**B**) pyrethrins, and synthetic pyrethrins I and II; (**C**) pyrethroid insecticides. Error bars indicate standard error of the mean (*n* = 5 repetitions), and asterisks denote significant differences from PI = 0 and 0% KD rate, according to a one-sample *t*-test with Bonferroni correction; * *p* < 0.05, ** *p* < 0.01.

The feeding deterrence of salty, bitter, and sour tastants was evaluated through choice feeding tests. As the concentrations of the tastants were increased in sucrose solutions, the mean PIs of the blowflies gradually decreased to −1.0, indicating complete feeding deterrence (Figure 3A). For each concentration of the compound, comparison of the mean PI with the value of 0 indicated that the MECs of NaCl, quinine, and tartaric acid for feeding deterrence were 250.0, 1.0, and 62.5 mM, respectively. KD rates did not increase with the increase in the concentrations of the tastants (Figure 3A).

Natural and synthetic pyrethrins exhibited concentration-dependent PI patterns, similar to those of unpalatable tastants (Figure 3B). The MECs of natural pyrethrins and synthetic pyrethrins I and II for feeding deterrence were all 62.5 μM. This is lower than the minimum concentration at which the KD rate increased significantly: by 16 times for natural pyrethrins and pyrethrin I, and more than 16 times for pyrethrin II. Allethrin, a synthetic pyrethroid with structural similarity to pyrethrin I, indicated an MEC identical to that of pyrethrin I: 16 times lower than the minimum concentration for KD activity (Figure 3C). Deltamethrin increased the KD rate at 3.9 μM, and the average values of PIs did not decrease significantly at concentrations below 62.5 μM. Thus, deltamethrin was distinct from natural and synthetic pyrethrins, as well as allethrin, because the latter compounds deterred the flies from feeding before inducing KD.

We estimated the individual intake of each test compound at the MEC (Table 1). Even at concentrations that decreased the PIs significantly, the blowflies ingested a substantial amount of tastants in the test. The average amounts of individual consumption were roughly correlated with the MECs for feeding deterrence. Although statistical significance was partly detected, quinine was the most potent of the three tastants in terms of the intake as well as the effective concentrations. The average individual consumption level of pyrethrins and pyrethroids at the MECs that elicited feeding deterrence or KD activity was below 200 ng. In natural and synthetic pyrethrins, having identical MECs for feeding deterrence, the average amount of individual consumption of natural pyrethrins and pyrethrin II was less than that of pyrethrin I, indicating that pyrethrin I had less feeding deterrence than natural pyrethrins and pyrethrin II. Although the natural pyrethrins had a higher MEC than that of deltamethrin, the individual intake of natural pyrethrins was not greater than that of deltamethrin. The blowflies ingested the same amount of allethrin as synthetic pyrethrin I.

### 3.3. Grooming of the Proboscis Characterizes Feeding Deterrence of Pyrethrins and Pyrethroids

The feeding deterrence of natural pyrethrins was associated with intensive grooming of the proboscis, initiated after interruption of feeding at the MEC (Video S1). Behavioral observations of the small population of blowflies indicated that grooming of the proboscis followed feeding in almost all visits to a food dish containing natural/synthetic pyrethrins or allethrin at the MEC (62.5 μM) for feeding deterrence (Table 1). In contrast, at the MEC of deltamethrin that initiated KD activity, the rate of feeding-induced proboscis grooming was merely 8% and did not differ statistically from the baseline (0%) of the untreated sucrose solution. For the three tastants, during feeding on sucrose solutions containing each taste substance at MEC, no grooming of the proboscis was observed. These results indicate that intense grooming of the proboscis initiated by feeding was associated with the feeding deterrence of natural/synthetic pyrethrins and allethrin.

The total amount of food consumed by individual flies was directly affected by the total number of visits to a food dish during the test period. In dual-choice tests of the three unpalatable tastants, the total number of visits to the food dish did not change, even when the blowflies were initially fed on a sucrose solution containing one of the tastants at the respective MEC for feeding deterrence (Table 1). Primary intake of natural/synthetic pyrethrins and allethrin at the MEC for feeding deterrence tended to decrease the total number of visits to the food dish; however, a statistically significant difference in the values for the untreated solution was observed only in natural pyrethrins and pyrethrin I. In contrast to the high rates of proboscis grooming, the KD rates at the end of the tests were 0% in pyrethrin II and allethrin. Natural pyrethrins and pyrethrin I showed KD rates of less than 10%, which were not significantly different from the rate of 0% for the untreated solution and three tastants. The primary intake of food containing deltamethrin at the MEC that caused KD activity did not affect the total number of visits to a food dish, although about half of the flies were knocked-down at the end of the 5 h tests.

When testing the MEC of the natural/synthetic pyrethrins and allethrin, careful observations showed that the flies often vomited between bouts of grooming of the proboscis and held the drop of the content at the tip of the labellum for a while, as seen in Video S1 (0:07–0:10 s; left individual).

## 4. Discussion

In this study, a choice-feeding paradigm of artificial diets was used to clarify the feeding deterrence of pyrethrins and pyrethroids. Our data show that natural/synthetic pyrethrins and pyrethroids, mixed in sucrose solution, reduced the feeding preference of the blowflies in a concentration-dependent manner, similar to those of unpalatable tastants (NaCl, quinine, and tartaric acid). The primary finding of this study is that natural pyrethrins and synthetic pyrethrins I/II exhibited antifeedant activities at sub-lethal concentrations in the choice feeding paradigm and were more potent than the three tastants in terms of MECs.

In the behavioral process for feeding deterrence, natural/synthetic pyrethrins and pyrethroids were clearly distinct from salty, bitter and sour tastants, inducing intensive grooming of the proboscis upon the interruption of feeding bouts. The characteristic proboscis grooming was initiated without paralyzing the legs and was sustained while keeping the capability for locomotion and flight (Video S1). Among natural/synthetic pyrethrins and pyrethroids, feeding-induced grooming of the proboscis was associated with the action of feeding deterrence; proboscis grooming occurred at the MECs for feeding deterrence in natural/synthetic pyrethrins and allethrin, but rarely occurred at the MECs for KD activity in deltamethrin. Natural/synthetic pyrethrins and allethrin commonly deterred feeding without significantly increasing the KD rate, and pyrethrin II and allethrin achieved feeding deterrence without reducing the number of visits to the food dish. These results consistently indicate that the significant reduction in the treated food intakes was not the consequence of the insecticidal action of the pyrethrins and pyrethroids, which immediately hampers and paralyzes the flies.

Regarding the action of pyrethroids on the peripheral nervous system of insects, a series of electrophysiological recordings have indicated that pyrethroid compounds and DDT analogs, each of which partially sharing the binding site of voltage-gated sodium channels, generate repetitive irregular firings to the labellar taste hairs of Australian blowflies and houseflies [45,46,47,48]. Similar neural excitation has also been observed in the neural activity of the antennal olfactory receptor neurons of moths treated with a pyrethroid insecticide [49]. Repetitive firing, or hyperexcitation, observed in peripheral nerves, is a major feature of the effects on neural excitability induced by natural pyrethrins and Type I pyrethroids, including allethrin, many of which have a structure partially similar to pyrethrins [4,5,7]. Thus, it is reasonable to conclude that the intake of natural/synthetic pyrethrins and allethrin immediately stimulates the oral gustatory systems of blowflies to induce irritating sensations that cause intensive grooming of the proboscis, which interrupts feeding. Notably, the mechanism of feeding deterrence involving an intrinsic pharmacological action on neuronal sodium channels is fundamentally different from those of antifeedants selectively bound to specific taste receptors, such as salty and bitter taste receptors. The irritating sensations underlying the feeding deterrence of pyrethrins and pyrethroids can be referred to as feeding irritation, in general terms, in contrast to contact irritation, which induces a repellent action on tarsal contact with pyrethroids [13,14,15].

For a description of the relative potency of feeding deterrence to KD activity, the test insecticides can be divided into two groups: one group included natural pyrethrins, synthetic pyrethrins I/II, and allethrin, wherein feeding deterrence preceded KD; the other group included deltamethrin, wherein KD preceded feeding deterrence. Interestingly, the grouping corresponds to the two types of effects on neural excitability; the former compounds are all Type I pyrethroids, which produce long trains of repetitive firing (hyperexcitation), and the latter, deltamethrin, is a Type II pyrethroid that contains the α-cyano-3-phenoxybenzyl moiety with more potent lethality than Type I pyrethroids. Type II pyrethroids generally do not induce repetitive firing but instead cause a use-dependent block of action potentials [50]. There are a few studies that demonstrate a slight activation of the insect central nervous system in a concentration- and time-specific manner [51]. The limited neural excitability in contrast with the potent activity of paralysis explains why deltamethrin rarely induced proboscis grooming, even at the MEC that caused KD. The pharmacological properties specific to natural pyrethrins and Type I pyrethroids may enable feeding deterrence at sub-lethal concentrations.

The potencies of KD and lethality of pyrethrins and pyrethroids depend on various properties such as intrinsic pharmacological activity, permeability, and tolerance for degradation [3]. Irritating sensations that are fast enough to interrupt continued feeding may especially reflect the strength of the intrinsic activity and permeability of the compound. We showed that the relative potencies of pyrethrins for feeding deterrence, based on the estimated individual food intakes at identical MECs, are: natural pyrethrins ≥ pyrethrin II > pyrethrin I. The order of the relative potencies has rarely been observed because the KD activities and lethality are generally more potent in pyrethrin I or II than in natural pyrethrins [52,53,54,55] with one exception [56]. At present, it is difficult to explain the reason for the potency of natural pyrethrins from the limited data on pyrethrins I and II. The remaining four components should be examined and compared with natural pyrethrins.

Natural pyrethrins and pyrethrin I reduced the number of visits and eventually elicited low rates of KD. Some level of neurotoxic effects caused by ingestion may contribute to the potency of the feeding deterrence of these compounds. Recent progress in our understanding of olfactory perception [57] and learning [58] of pyrethrins and pyrethroids suggests another possibility: that flies can associate the olfactory/taste cues of the insecticides with the negative experience brought on by the toxic effects, to avoid further feeding. Considering the potential of these causal mechanisms, the net feeding deterrence based on oral sensory stimulation should be investigated by an assay system, such as a manual capillary feeding assay [34], which supports manipulation of the feeding of a single restrained fly and the involvement of sensory organs.

In conclusion, we observed that natural pyrethrins and pyrethrins I/II act as potent feeding deterrents at sub-lethal concentrations for blowflies. Our results suggest that the major mode of action for feeding deterrence of natural pyrethrins and pyrethrins I/II, though probably operating on the oral gustatory system of the insects, is distinct from those of salty, bitter, and sour tastants. As most insect species carry only one sodium channel gene [59], natural pyrethrins have the potential for feeding deterrence on a variety of herbivorous insects that are susceptible to pyrethrins and pyrethroid insecticides, including the species hosted by the pyrethrum daisy [11].Taken together with the fact that natural pyrethrins are more potent than two of their most insecticidal constituents, pyrethrins might have evolved to enhance anti-feedance as a mixture, in order to defend pyrethrum plants effectively by rapidly deterring herbivorous insects from continuous feeding. To verify this hypothesis, future studies on individual species with different feeding types are needed to investigate how, if at all, pyrethrins deter herbivorous insects from sucking/piercing or chewing. Furthermore, an electrophysiological approach should be used to determine how the intake of pyrethrins stimulates the oral gustatory system of insects. *Drosophila* flies and blowflies will be useful model organisms in such physiological studies.

## Figures and Tables

**Table 1:** *Phormia regina* feeding behavior and knockdown rates at a minimum effective concentration (MEC) of pyrethrins, pyrethroids, and unpalatable tastants.

Test Compounds		MEC ^§^ (Effect)	Estimated Individual Intake (ng)	Behavior at the MEC ^£^		
				Number of Visits	% of Proboscis Grooming	KD Rate (%)
Tastants	NaCl	250 mM (FD)	137742 ± 28097 ^e^	3.57 ± 0.39 ^a^ (*n* = 21)	0 ^a^	0 ^a^
	Quinine	1 mM (FD)	602 ± 169 ^d^	3.70 ± 0.47 ^a^ (*n* =23)	0 ^a^	0 ^a^
	Tartaric acid	62.5 mM (FD)	20947 ± 6658 ^d^	3.73 ± 0.30 ^a^ (*n* = 22)	0 ^a^	0 ^a^
Insecticides	Natural pyrethrins	62.5 μM (FD)	39.2 ± 16.7 ^a^	1.75 ± 0.21 ^bc^ (*n* = 24)	90.2 ^b^	2.9 ^a^
	Synthetic pyrethrin I	62.5 μM (FD)	199.9 ± 34.2 ^c^	2.18 ± 0.21 ^c^ (*n* = 22)	95.5 ^b^	10.0 ^a^
	Synthetic pyrethrin II	62.5 μM (FD)	75.8 ± 16.7 ^ab^	2.78 ± 0.25 ^ab^ (*n* = 27)	97.4 ^b^	0 ^a^
	Allethrin	62.5 μM (FD)	141.4 ± 17.6 ^bc^	3.13 ± 0.27 ^ab^ (*n* = 32)	90.3 ^b^	0 ^a^
	Deltamethrin	3.9 μM (KD)	12.5 ± 2.6 ^a^	3.36 ± 0.47 ^a^ (*n* = 22)	7.95 ^a^	51.2 ^b^
Control solution	Sucrose 0.1 M (incl. DMSO 1%)			4.08 ± 0.41 ^a^ (*n* = 24)	0 ^a^	0 ^a^

^§^ Minimum effective concentration (MEC) for feeding deterrence (FD) or knockdown activity (KD) was defined as the minimum concentration at which the value of the feeding preference index significantly decreased or the KD rate increased, respectively, in a 5 h dual-choice feeding assay. The average individual intake of a compound was estimated by dividing the weight of the test compound present in the ingested sucrose solution, at the MEC, by the number of flies released into the cage. Mean ± SE are presented (5 repetitions). The values with the different superscript letters a–e are significantly different (*p* < 0.05). ^£^ Feeding behaviors of the flies were recorded in the first 30 min of the dual-choice feeding test at the MEC of a test compound (*n* = 45 in each). In an analysis, the number of visits to food dishes were counted for the individuals who first visited the treated food dish. The Tukey’s method was used for multiple comparisons of the mean values within a group (mean ± SE). The frequency of occurrence of proboscis grooming during visits to the treated food dishes (*n* = 44–103), and the rate of occurrence of KD at the end of the test were also obtained. The Fisher’s exact test, with *p*-values adjusted using the Holm’s method, was used for multiple comparisons within a group. The values with the different superscript letters in each column are significantly different (*p* < 0.05).

## Data Availability

The datasets generated and/or analyzed during the current study are available from the corresponding author upon reasonable request.

## Supplementary Materials

The following supporting information can be downloaded at: https://www.mdpi.com/article/10.3390/insects13080678/s1, Video S1: Irritant response to the oral intake of natural pyrethrins. Blowflies stop feeding on sucrose solution mixed with natural pyrethrins (62.5 μM), and start grooming their proboscises with their forelegs. Flies vomit during the irritant behavior (0:07–0:10 s; left individual) prior to flying away from the feeding dish.
